# The Psychological Impact and Influencing Factors during Different Waves of COVID-19 Pandemic on Healthcare Workers in Central Taiwan

**DOI:** 10.3390/ijerph191710542

**Published:** 2022-08-24

**Authors:** Teh-Kuang Sun, Li-Chuan Chu, Chun Hui

**Affiliations:** 1 Department of Internal Medicine, Chung Kang Branch, Cheng Ching Hospital, Taichung City 40764, Taiwan; 2 School of Health Policy and Management, Chung Shan Medical University, Taichung City 40201, Taiwan; 3 Department of Medical Education, Chung Shan Medical University Hospital, Taichung City 40201, Taiwan

**Keywords:** COVID-19, healthcare workers, psychological impact

## Abstract

Background: This study aims to explore differences of psychological impact and influencing factors that affected Taiwanese healthcare workers (HCW) during the first and second wave of COVID-19. Methods: a cross sectional survey of first-line HCW during November 2021 to February 2022: 270 paper questionnaires were issued and the valid response rate was 86% (231). For statistical analysis, descriptive statistics, Pearson correlation, and multivariate linear regression were used. Results: regardless of the wave of the pandemic, nearly 70% of HCW had anxiety, nearly 60% felt depressed, half of them suffered from insomnia, and one in three felt insufficient social support, which means a high level of loneliness. With an increased number of infected patients during the second wave, HCW felt significant changes of workload and schedule, with higher concern over risk of infection, and these factors induced higher levels of anxiety, but they manifested better satisfaction over public health policies and information provided by hospitals and governments. Changes of working schedules or duties positively relate to levels of anxiety and insomnia. The risk of infection causes anxiety, depression, and insomnia. Workplace relationships significantly relate to depression and loneliness. A negative family support causes an adverse psychological impact. Conclusions: the pandemic has a negative psychological impact on HCW. Early recognition of significant influencing factors, providing psychological support and therapy, are helpful strategies for reducing the adverse psychological effects.

## 1. Introduction

The coronavirus disease (COVID-19) has spread worldwide since the end of 2019; it has been affecting millions of people, which exceeds in number and scale severe acute respiratory syndrome (SARS) and Middle East respiratory syndrome (MERS), causing a heavy burden on public health systems globally, consequently bringing impacts to healthcare workers (HCW) in an unprecedented way.

Since its first imported case in January 2020, Taiwan experienced three definite waves of COVID-19 infections, different from other countries, with no new diagnosed cases between. Taiwan had a relatively lower number of confirmed cases and deceased patients in its first wave during January to May of 2020, followed by nearly 10 months of zero cases until April of 2021, when multiple chains of infection spread rapidly. It was a second wave of infection. By the end of August of 2021, there were more than 15,000 confirmed cases and over 800 deceased cases (Table 1). As most of the newly diagnosed patients were in the northern region of the country, the Taiwanese center of disease control (CDC) strategically deferred patients to medical centers and regional hospital in central and south Taiwan to unload the burden on hospitals in the northern regions. The number of new diagnosed cases dropped to a single digit by the beginning of September 2021, followed by a period of nearly no new cases until January of 2022, when the Omicron variant appeared, causing the third wave of infection.

From experience of previous pandemics, these situations can cause detrimental psychological effects on HCW [1,2,3]. Taiwan’s experience with SARS in 2003 demonstrated that nearly 70% of HCW had mental health burdens [4]. In the early stage of the COVID-19 pandemic in Taiwan, a web-based survey of HCW from March to April of 2020 demonstrated that 40.3% of responders felt burnt out, with a significant level of anxiety in 78.1%, and 45.5% complained of depression [5]. 

It is well-known that psychological problems in HCW during a pandemic are multifactorial: some factors are intrinsic to HCW, such as, for example, gender, occupation, education level, position, seniority, [2,3,6,7], frontline HCW [8], marital status/living conditions [9], use of tobacco/alcohol [10,11], and if they ever have any chronic disease or mental disease [7]. Other factors are extrinsic to HCW, such as work loading and shift changes [1,2,12], availability of personal protection equipment (PPE) and/or medical resources [6,13], risk of infection [1,2,3], workplace relationships between leaders and co-workers [7], social pressure and stigmatization [14], family support [9], and transparency/accuracy of public health policy and information from governments and hospitals [15,16]. 

Most of studies conducted during the COVID-19 pandemic had focused on the detrimental psychological effects of the pandemic and its related factors on HCW. Some studies had focused on a single second wave of infection [17,18]. A study comparing two waves of infection in India reported that HCW were less affected by psychological impacts during the second wave [19], but two Italian studies comparing psychological stress in the first and second waves reported no significant differences between the two waves [20,21]. These findings leave questions such as, if in different countries, with a variety in backgrounds or influencing factors, how can the negative psychological effects and severity in HCW during different waves of the pandemic differ?

In this study, we aim to investigate: 1. differences of severity of psychological impacts between the first and second waves of the pandemic in HCW in Taiwan, and 2. analysis of extrinsic factors to see how differently they manifested in both stages of the infection, and how they affected the negative psychological effects on Taiwanese HCW.

## 2. Materials and Methods

Study design and participants: a cross-sectional study was conducted in a regional teaching hospital in central Taiwan between 1 November 2021 and 28 February 2022, after two waves of infection in Taiwan. Participants were first-line HCW who actively cared and treated suspected or confirmed COVID-19 cases, including physicians, nurses, physician assistants (PA), respiratory therapists (RT), and radiological and laboratory medical technical assistants (MTA), as well as pharmacists. Staffs of emergency rooms, intensive care units, and isolation rooms where included, with professionals who are specialized in infection disease, pneumology, intensive care, and emergency medicine. Two hundred and seventy paper questionnaires were issued.

The contents of the questionnaire consisted of three parts: the first part includes socio-demographic parameters (age, gender, occupation, years of professional experience, marital status, living alone or not, education level, previous working experience on infection disease, history of chronic disease and psychiatric disease, and current use of alcohol or cigarettes); these were defined as intrinsic factors.

The second part of the questionnaire consisted of an evaluation of psychological impact. We used the Hospital Anxiety and Depression Scale (HADS) [22]; this includes eight items for anxiety and seven items for depression. Using a four-point Likert scale, the maximum score is 21, a score under 8 is considered normal, a score of 8 to 10 indicates a borderline level, and 11 to 21 indicates an abnormal level. Its Cronbach’s α coefficient was 0.91. 

For evaluation of insomnia, we used Insomnia Severity Index (ISI) [23], which includes seven items; using a five-point Likert scale, a maximum score was 21 and the level of insomnia was defined as follows: a score of 8 to 14 indicates a subthreshold level, 15 to 21 indicates a moderate level, 22 to 28 indicates severe insomnia, and its Cronbach’s α coefficient was 0.91. To reflect the level of loneliness, we used the Oslo social support scale (OSSS–3) [24], which includes three items; using a four- to five-point Likert scale, a score of 3–8 indicates poor support, 9–11 indicates a moderate level of support, and 12–14 indicates strong social support. OSSS–3’s Cronbach’s α coefficient in this study was 0.77.

The third part of the questionnaire evaluates seven aspects of working conditions and potential problems, defined as extrinsic factors. We used a four-point Likert scale. These factors include: changes of workload and schedules (five items, ranging from “no” to “very frequent”), sufficiency of PPE and medical supplies (four items, ranging from “not enough” to “very complete”), concerns over the risk of infection (four items, ranging from “not worried” to “very worried”), working place relationships (four items, ranging from “none at all” to “very good”), social pressure (three items, ranging from “none at all” to “very frequent”), family support (two items, ranging from “none at all” to “very supportive”), and public health policy and information accuracy and transparency (five items, ranging from “none at all” to “very complete”). The Cronbach’s α coefficient was 0.77 to 0.92, which indicates an acceptable level of internal consistency.

Statistical analysis: the baseline socio-demographic characteristics and psychological impacts were analyzed with descriptive statistics.

To compare differences of psychological impacts during the first wave and second wave of infection, we used a paired sample *t*-test. The Pearson correlation test was applied to extrinsic factors with the intention to analyze their relation to psychological effects; then, we compared differences of extrinsic factors during the first wave and second wave of infection with a paired sample *t*-test. Multiple linear regression analysis on extrinsic factors was employed to evaluate their effect on psychological impact during the most affected wave of infection.

Statistical analysis was performed in an IBM SPSS Statistics version 26, with a two-tailed *p* value of <0.05 indicating significance.

## 3. Results

Two hundred and seventy paper questionnaires were issued, 39 incomplete questionnaires were excluded, and the complete response rate was 85.55%. The mean age of participants was 35.85 years (SD = 9.76). The mean years of professional experience was 12.06 (SD = 8.82) and 75.8% were women (n = 175). Nearly 70% were university graduates (69.7%, n = 161). Nurses composed 55.4% (n = 128) of the total sample size. Nearly half of responders were married (50.2%, n = 116) and most of them live with family members (77.5%, n = 179). Very few responders have chronic disease, psychiatric disease, or a habit of alcohol–cigarettes use (Table 2). 

Levels of anxiety, depression, insomnia, and social support during the first wave and second wave of infection were analyzed.

In both the first and second waves of the pandemic, more than 40% of HCW suffered from abnormal levels of anxiety, nearly 30% had abnormal levels of depression, around 15% of them suffered from moderate to severe insomnia, and one in three had low levels of social support (Table 3). 

A paired sample *t*-test to compare psychological impacts during the first and second waves of infection was performed. Compared to the first wave, anxiety is significantly more severe during the second wave of infection (*M* = 10.09, *SD* = 5.71 vs. *M* = 10.65, *SD* = 5.11, *p* = 0.049), without a significant difference in the levels of depression, insomnia, and social support between the first and second waves (Table 4). 

Once we compared both waves of infections and their differences, based on the findings of the second wave, for further analysis and understanding of the relationship between extrinsic factors and negative psychological impacts, we used Pearson correlation (Table 5).

Changes of workload and schedules (*r* = 0.43, *p* < 0.001), concerns over risk of infection (*r* = 0.54, *p* < 0.001), and social pressure (*r* = 0.34, *p* < 0.001) were positively related to anxiety. Sufficiency of personal protection equipment and medical supplies (*r* = −0.29, *p* < 0.001), working place relationships (*r* = −0.22, *p* < 0.001), family support (*r* = −0.16, *p* = 0.01), and public health policy and information accuracy and transparency (*r* = −0.29, *p* < 0.001) were negatively related to anxiety. Extrinsic factors that are positively related to depression are changes of workload and schedules (*r* = 0.41, *p* < 0.001), concerns over risk of infection (*r* = 0.45, *p* < 0.001), and social pressure (*r* = 0.23, *p* < 0.001). Factors that negatively related to depression are sufficiency of personal protection equipment and medical supplies (*r* = −0.26, *p* < 0.001), working place relationships (*r* = −0.34, *p* < 0.001), family support (*r* = −0.27, *p* < 0.001), and public health policy and information accuracy and transparency (*r* = −0.31, *p* < 0.001).

In relation to insomnia, changes of workload and schedules (*r* = 0.44, *p* < 0.001), concerns over risk of infection (*r* = 0.38, *p* < 0.001), and social pressure (*r* = 0.25, *p* < 0.001) were positively related to insomnia. Sufficiency of personal protection equipment and medical supplies (*r* = −0.29, *p* < 0.001), working place relationships (*r* = −0.28, *p* < 0.001), family support (*r* = −0.21, *p* = 0.001), and public health policy and information accuracy and transparency (*r* = −0.29, *p* < 0.001) were negatively related to insomnia.

Extrinsic factors that positively related to social support are sufficiency of personal protection equipment and medical supplies (*r* = 0.28, *p* < 0.001), working place relationships (*r* = 0.39, *p* < 0.001), family support (*r* = 0.37, *p* < 0.001), and public health policy and information accuracy and transparency (*r* = 0.28, *p* < 0.001). Factors that negatively related to social support were changes of workload and schedules (*r* = −0.13, *p* = 0.04).

Analysis using a paired sample *t*-test comparing the first and second waves on extrinsic factors showed changes of workload and schedules were more significant during the second wave (*M* = 1.18, *SD* = 0.70 vs. *M* = 1.04, *SD* = 0.67, *p* < 0.001), as well as increased concerns over risk of infection (*M* = 1.56, *SD* = 0.75 vs. *M* = 1.47, *SD* = 0.74, *p* < 0.05). Public health policy and information accuracy and transparency were considered as having improved during the second wave (*M* =1.82, *SD* = 0.55 vs. *M* = 1.77, *SD* = 0.52, *p* < 0.01) (Table 6).

In multivariate correlations analysis, increases in workload and schedules (*β* = 0.13, *p* = 0.043), concerns over risk of infection (*β* = 0.42, *p* < 0.001), and lower levels of family support (*β* = −0.15, *p* = 0.006) *worsen* *levels of anxiety*. Concerns over risk of infection (*β* = 0.39, *p* < 0.001), poor working place relationships (*β* = −0.19, *p* < 0.01), and insufficient family support (*β* = −0.22, *p* < 0.001) are related to depression.

Insomnia can be aggravated by changes in workload and schedules (β = 0.23, *p* = 0.001), more concerns over risk of infection (β = 0.22, *p* = 0.001), and poor family support (β = −0.16, *p* = 0.008). Better levels of working place relationships (β = 0.27, *p* < 0.001) and family support (β = 0.30, *p* < 0.001) and better the perception of social support indicates a lower level of loneliness (Table 7).

## 4. Discussion

Our study had demonstrated that the COVID-19 pandemic causes negative psychological impacts on HCW regardless of the stages/waves of the infection or the severity of the pandemic. Despite the low number of confirmed cases during the first wave of the COVID-19 pandemic in Taiwan, more than 40% of our first-line HCW had abnormal levels of anxiety, nearly 30% had abnormal levels of depression, approximately 15% had moderate to severe insomnia, and more than 30% felt loneliness. During the second wave, even though the country had nearly 10 months of zero cases, enough time on either the institutional level or personal level to prepare for the possibility of a larger-scale second hit, which had occurred during April–August of 2021, a significant higher level of anxiety was observed in HCW during this period, compared to the first wave. The levels of depression, insomnia, and loneliness, reflected by social support, remained similar in both waves.

Based on Pearson correlation analysis, all seven extrinsic factors investigated in this study, either positively or negatively, had influenced a negative psychological impact on HCW included in this study, with some of them being much more significant. 

Increased changes of workload and schedules had a significant adverse psychological effect, particularly increasing levels of anxiety and insomnia during the second wave of infection in Taiwan, while the pandemic became exacerbated. The work burden, psychological distress, and insomnia during COVID-19 are interrelated and multifactorial [25]. A study conducted by Boudreau et al. proved that shift work causes sleep disorders that consequently negatively disturb the moods of HCW [26]. More frequent is the shift of work schedule, especially alternating day–night shifts, when more occurrence of shift work sleep disorder (SWSD) is observed, contributing to the severity of anxiety and depression [27]. A study based on a university hospital in Portugal in 2020 during the COVID-19 pandemic proved that working overtime is one of the main factors causing anxiety, depression, and psychological stress; more overtime, the worse the negative psychological impact [12]. Work burden as a stress factor should be addressed, as increased workload and schedule shift worsens the severity of anxiety, altering sleeping pattern, in combination, they could induce abnormal hypothalamo–pituitary–adrenal secretory activity with subsequent deterioration of sleep disturbances [28], which further weakens the immune system, increasing even more the risk of infection [29].

With a rapid increase in the numbers of infected patients, our frontline HCW were more concerned with being infected, or being the source of infection of coworkers and family members, and these concerns significantly affected the anxiety, depression, and insomnia of HCW in this study. The anxiety over the risk of infection for HCW is not only related to increases in the numbers of infected patients, but also because the excess of workload/schedule shift altering sleeping patterns may contribute to an altered immune system, as mentioned before. As a consequence, during the second wave, the number of infected HCW was in fact increased. This is a real threat to our HCW [30]. Studies showed that this is one of main stress factors to HCW during previous major pandemics [1,31]. From the experience of SARS in 2003, study of the post-pandemic stage proved that working in a high-risk environment and direct contact with infected patients, with fear of being a source of infection to family, caused a very high level of psychological stress that persisted even after the SARS pandemic had waned [32]. Observations from studies of the early stage of the COVID-19 pandemic found that worries over the risk of infection on HCW themselves, and, more, to colleagues or family members, significantly exacerbates the levels of anxiety and depression of frontline HCW [7,33].

As HCW face an immense increase in duties during pandemics, well-functioning teamwork is essential, workplace relationships play an important role as an influencing factor on the psychological impacts of HCW. Our study showed that the better the workplace relationships, the better the sense of social support, and the lower the level of depression. A study in Germany showed that a lack of trust between co-workers of a medical team is a risk factor for anxiety and depression. Confidence and support from the leadership is essential, encouraging team members to manifest their needs and challenges, listen to their opinions and advice, and provide mutual support to find solutions together, and are very helpful strategies to decrease the psychological impacts of HCW [34,35].

In our study, more than 70% of our HCW felt that the public demands too much from them, showing certain levels of discrimination or disrespect. Despite that social pressure seems to correlate positively to anxiety, depression, and insomnia in our study, statistically it did not show significant negative psychological impact on HCW. Possible explanations for this result are good family support and adequate working place relationships: more than 70% of our HCW responded as having sufficient support from their family, and nearly 80% responded as having adequate working place relationships with their co-workers and leaders. Several studies concluded that satisfaction over family support and working place relationships helps in ameliorating negative psychological effects from social pressure [34,36,37]. 

Family support is one of the essential factors for the resilience of HCW during a pandemic. Our study proved that the less the family support, the worse the level of anxiety, depression, and insomnia, and the better the family support, the better the sense of social support, which means fewer feelings of loneliness. Chen et al., in their study of Taiwanese HCW during SARS, proved that good family support helped in reducing the psychological stress and improving resilience during their fight against the pandemic [38]. A study from Korea concluded that one of the main factors related to psychological fatigue in HCW during MERS is a lack of family support [39]. Our study also showed that the better the family members understand the job of HCW, the better they felt supported; this finding is compatible to other studies [34,36].

Sufficiency of PPE and medical supplies and public health policy/information accuracy and transparency are two factors that did not affect HCW in our study.

After a temporary shortage of PPE and medical supplies during the first wave, the Taiwanese government had responded efficiently by providing medical resources to hospitals in charge, so more than half of our HCW considered that the government and hospital had provided enough PPE and medical supplies. Sampaio et al., in their study, concluded that sufficient provision of PPE and medical supplies and securing their quality significantly reduces anxiety, depression, and stress in frontline HCW [12]. 

In this study, our HCW responded with a high level of satisfaction about public health policy and information accuracy and transparency, as more than 70% of them responded “complete” or “very complete” on this issue. It is crucial to provide transparent and concrete information and policies [1]. A better informed and trained HCW is more confident in their duties, and consequently has less stress. The Taiwanese CDC has held press conferences daily since the very beginning of the pandemic, providing information and guidelines and announcing policies; this is very helpful to avoid misinformation and fake news, and avoid chaos and panic, which coincides with the findings of previous studies [16]. A study from China concluded that uncertainties in public health policies and guidelines reduces work efficiency and causes psychological stress for HCW [15]. Japanese studies also suggested that transparency of information from the government and hospitals in combination with adequate on-the-job training significantly reduces the anxiety and stress of HCW during a pandemic [6,13].

This study had some limitations: first, this study was based in a single regional healthcare system in central Taiwan, so a study of a larger scale on the cross-regional or national level would help to further understand HCW’s psychological impact in Taiwan. Despite of this limitation, this study can provide information, references, and guidance for future studies. Second, as we could never predict how the pandemic would develop in the country, a 10-month interval between the first wave and second wave was not expected. The intention of comparing the psychological impacts on HCW in two different waves surged after the second wave had been waning, so the study was designed as a cross-sectional study, conducted in the time that the second wave had ended. To reduce recall bias, a longitudinal study, which would reflect the psychological impact in real time, could be considered for future studies. 

In summary, during the COVID-19 pandemic in Taiwan, HCW were significantly affected by negative psychological impacts such as anxiety, depression, insomnia, and loneliness, regardless of the wave of infection. A significant higher level of anxiety was observed during the second wave of COVID-19 in Taiwan, when the number of infected patients had surged rapidly and, consequently, the workload increased and changes of working schedules varied significantly. HCW became more concerned over being infected or being a source of infection during the second wave. By the same period, HCW demonstrated more confidence and satisfaction in public health policy and information accuracy and transparency than during the first wave. Increased changes in workload and working schedules, concerns over the risk of infection, workplace relationships, and family support were significant influencing factors on the negative psychological impacts.

## 5. Conclusions

COVID-19 is the major and most recent pandemic that human beings have faced in the 21st century but would not be the last. By the time of redaction, many countries continue to be under the pressure of COVID-19 Omicron variants such as BA.4 and BA.5, and Taiwan is experiencing the third wave of the COVID-19 pandemic; meanwhile, monkeypox has been declared by the WHO to be a public health emergency of international concern.

The challenge continues. Understanding the influencing factors that affect HCW psychologically is helpful and essential for building mental health strategies, which should be timely, flexibly, and holistically responsive to protect our HCW [1,34,36,40]. Administrative efforts to reduce workload and to adjust working schedules, providing medical resources to reduce risk of infection, and creating a supportive working environment and good family support are important measures to protect our frontline HCW. 

## Figures and Tables

**Table 1:** Number of confirmed cases and deceased cased in comparison to neighboring countries of Taiwan such as Japan and Korea.

	By 31 May 2020	By 31 August 2021	By 31 May 2020	By 31 August 2021
	Confirmed Cases (No)	Deceased Cases (No)	Confirmed Cases (No)	Deceased Cases (No)
Taiwan	442	7	15,995	835
Japan	16,741	898	1,494,372	16,065
South Korea	11,503	271	253,445	2292

Source: COVID-19 dashboard. https://covid–19.nchc.org.tw/index.php?language=en, accessed on 30 July 2022.

**Table 2:** Sociodemographic characteristics of the respondents.

Mean Age (years) (SD)	35.85 (9.76)	Professional Experience (years) (SD)	12.06 (8.82)
	No (%)		No (%)
**Gender**		**Marital status**	
Male	56 (24.2)	Single	112 (48.5)
Female	175 (75.8)	Married	116 (50.2)
**Education level**		Divorced or separated	3 (1.3)
Technical school	55 (23.8)	**Underage children**	
University	161 (69.7)	Yes	93 (40.3)
Post–graduate	15 (6.5)	No	138 (59.7)
**Occupation**		**Livnig status**	
Nurse	128 (55.4)	Living alone	52 (22.5)
PA and RT	31 (13.4)	Living with family	179 (77.5)
Physician	23 (10.0)	**Personal habits**	
MTA and pharmacist	49 (21.2)	Smoking	8 (3.5)
**Service setting**		Alcohol	11 (4.8)
Ward	82 (35.5)	No	212 (91.7)
Intensive care unit	63 (27.3)	**Chronic disease**	
Emergency room	37 (16.0)	Yes	28 (12.1)
MTA units and pharmacy	49 (21.2 )	No	203 (87.9)
**Previous professional experience in pandemic/epidemics**		**Pre-pandemic** **psychiatric illness**	
Yes	112 (48.5)	Yes	8 (3.5)
No	119 (51.5 )	No	223 (96.5)

**Table 3:** Level of anxiety, depression, insomnia, and social support during first wave and second wave.

	1st Wave	2nd Wave		1st Wave	2nd Wave
**Anxiety** No( %)			**Depression** No (%)		
No	77 (33%)	64 (28%)	No	121 (47%)	91 (39%)
Borderline	56 (24%)	60 (26%)	Borderline	62 (24%)	61 (27%)
Abnormal	98 (43%)	107 (46%)	Abnormal	76 (29%)	79 (34%)
**Insomnia** No (%)			**Social support** No (%)		
No	114 (49%)	102 (44%)	Poor support	73 (32%)	70 (30%)
Subthreshold	83 (36%)	100 (43%)	Moderate support	104 (45%)	104 (45%)
Clinically moderate	26 (11%)	20 (9%)	Strong support	54 (23%)	57 (25%)
Clinically severe	8 (4%)	9 (4%)			

**Table 4:** Paired sample *t*-test to compare psychological impact during first and second wave of infection.

	Mean(SD)		df	*t*	*p*	*d*
	First Wave	Second Wave				
**Anxiety**	10.09 (5.717)	10.65 (5.119)	230	−1.975	0.049	−0.103
**Depression**	8.32 (4.285)	8.63 (4.206)	230	−1.595	0.112	−0.073
**Insomnia**	8.30 (6.085)	8.49 (5.880)	230	−0.958	0.339	−0.032
**Social support**	9.73 (2.467)	9.82 (2.446)	230	−1.449	0.149	−0.037

**Table 5:** Pearson correlation: extrinsic factors, anxiety, depression, insomnia and social support.

	1	2	3	4	5	6	7	8	9	10	11
1. **Changes of workload and schedules**	(0.86)										
2. **Sufficiency of** **PPE** **and medical supplies**	−0.325 ***	(0.90)									
3. **Concerns over risk of infection**	0.468 ***	−0.268 ***	(0.92)								
4. **Working place relationships**	−0.334 ***	0.510 ***	−0.105	(0.89)							
5. **Social pressure**	0.324 ***	−0.263 ***	0.388 ***	−0.135 *	(0.89)						
6. **Family support**	−0.152 *	0.178 **	0.066	0.284 ***	0.032	(0.83)					
7. **Public health policy and information accuracy and transparency**	−0.408 ***	0.690 ***	−0.206 **	0.559 ***	−0.202 **	0.234 ***	(0.87)				
8. **Anxiety**	0.437 ***	−0.294 ***	0.547 ***	−0.222 **	0.341 ***	−0.169 *	−0.298 ***	(0.90)			
9. **Depression**	0.410 ***	−0.263 ***	0.458 ***	−0.341***	0.236 ***	−0.271 ***	−0.310 ***	0.600 ***	(0.84)		
10. **Insomnia**	0.445 ***	−0.292 ***	0.382 ***	−0.287 ***	0.259 ***	−0.219 **	−0.295 ***	0.475 ***	0.519 ***	(0.91)	
11. **Social support**	−0.134 *	0.281 ***	−0.125	0.390 ***	−0.094	0.374 ***	0.284 ***	−0.173 **	−0.247 ***	−0.241 ***	(0.77)
*Mean*	1.18	1.51	1.56	2.01	1.60	2.17	1.83	10.64	8.6	8.5	9.82
*SD*	0.70	0.69	0.75	0.57	0.74	0.75	0.55	5.14	4.23	5.88	2.44

Chronbach’s α coefficient value is showed on the diagonal line. *SD* = standard deviation. * *p* < 0.05, ** *p* < 0.01, *** *p* < 0.001.

**Table 6:** Paired *t*-test comparing extrinsic factors during first and second wave.

	Mean(*SD*)		*df*	*t*	*p*	*d*
	First Wave	Second Wave				
**Changes of workload and schedules**	1.04 (0.67)	1.18 (0.70)	230	−4.18	<0.001	−0.204
**Sufficiency of PPE and medical supplies**	1.49 (0.66)	1.51 (0.69)	230	−0.685	0.494	−0.030
**Concerns over risk of infection**	1.47 (0.74)	1.56 (0.75)	230	−2.425	0.016	−0.121
**Working place relationships**	2.03 (0.58)	2.01 (0.56)	230	0.603	0.54	0.035
**Social pressure**	1.55 (0.74)	1.59 (0.74)	230	−1.604	0.11	−0.054
**Family support**	2.14 (0.76)	2.17 (0.75)	230	−1.129	0.26	−0.040
**Public health policy and information accuracy and transparency**	1.77 (0.52)	1.82 (0.55)	230	−2.639	0.009	−0.093

**Table 7:** Multiple linear regression analysis on extrinsic factors during second wave of infection.

	Anxiety			Depression		
	*B*	*SE B*	*β*	*B*	*SE B*	*β*
1. **Changes of workload and schedules**	0.972 *	0.478	0.133 *	0.688	0.402	0.114
2. **Sufficiency of PPE and medical supplies**	−0.125	0.563	−0.017	0.431	0.474	0.071
3. **Concerns over risk of infection**	2.929 ***	0.431	0.429 ***	2.195 ***	0.363	0.391 ***
4. **Working place relationships**	−0.232	0.603	−0.0026	−1.444 **	0.508	−0.193 **
5. **Social pressure**	0.790	0.403	0.114	0.191	0.339	0.034
6. **Family support**	−1.057 **	0.381	−0.154 *	−1.260 **	0.321	−0.223 ***
7. **Public health policy and information accuracy and transparency**	−0.651	0.733	−0.070	−0.497	0.617	−0.065
*R^2^*	0.390			0.362		
*Adj R^2^*	0.371			0.342		
*F*	20.34			18.5		
*df*	(7223)			(7223)		
	**Insomnia**			**Social Support**		
	***B***	***SE B***	***β***	***B***	***SE B***	***β***
1. **Changes of workload and schedules**	1.982 **	0.589	0.237 **	0.368	0.251	0.106
2. **Sufficiency of PPE and medical supplies**	−0.418	0.694	−0.049	0.202	0.296	0.057
3. **Concerns over risk of infection**	1.784 **	0.531	0.229 **	−0.434	0.227	−0.134
4. **Working place relationships**	−1.000	0.743	−0.096	1.186 ***	0.317	0.275 ***
5. **Social pressure**	0.557	0.497	0.070	−0.092	0.212	−0.28
6. **Family support**	−1.263 **	0.470	−0.161 **	0.992 ***	0.200	0.305 ***
7. **Public health policy and information accuracy and transparency**	−0.129	0.904	−0.012	0.130	0.386	0.029
*R^2^*	0.292			0.251		
*Adj R^2^*	0.270			0.227		
*F*	13.13			10.67		
*df*	(7223)			(7223)		

N = 230. * *p* < 0.05, ** *p* < 0.01, *** *p* < 0.001.

## Data Availability

Not applicable.
